# Practices and intravascular catheter infection during on- and off-hours in critically ill patients

**DOI:** 10.1186/s13613-021-00940-3

**Published:** 2021-10-29

**Authors:** Niccolò Buetti, Stéphane Ruckly, Jean-Christophe Lucet, Arthur Mageau, Claire Dupuis, Bertrand Souweine, Olivier Mimoz, Jean-François Timsit

**Affiliations:** 1 University of Paris, INSERM, IAME, 75006 Paris, France; 2 AP-HP, Infection Control Unit, Bichat- Claude Bernard University Hospital, 46 rue Henri Huchard, 75877 Paris Cedex, France; 3 Medical and Infectious Diseases Intensive Care Unit, AP-HP, Bichat-Claude Bernard University Hospital, 46 rue Henri Huchard, 75877 Paris Cedex, France; 4 Medical ICU, Gabriel-Montpied University Hospital, Clermont-Ferrand, France; 5 Services des Urgences Adultes and SAMU 86, Centre Hospitalier Universitaire de Poitiers, 86021 Poitiers, France; 6 Université de Poitiers, Poitiers, France; 7 Inserm U1070, Poitiers, France; 8 Infection Control Program and WHO Collaborating Centre On Patient Safety, University of Geneva Hospitals and Faculty of Medicine, Geneva, Switzerland

**Keywords:** Catheter, Catheter-related bloodstream infection, Bloodstream infection, ICU, Off-hours, On-hours

## Abstract

**Background:**

The potential relationship between intravascular catheter infections with their insertion during weekend or night-time (i.e*.*, off-hours or not regular business hours) remains an open issue. Our primary aim was to describe differences between patients and catheters inserted during on- *versus* off-hours. Our secondary aim was to investigate whether insertions during off-hours influenced the intravascular catheter infectious risks.

**Methods:**

We performed a post hoc analysis using the databases from four large randomized-controlled trials. Adult patients were recruited in French ICUs as soon as they required central venous catheters or peripheral arterial (AC) catheter insertion. Off-hours started at 6 P.M. until 8:30 A.M. during the week; at weekend, we defined off-hours from 1 P.M. on Saturday to 8.30 A.M. on Monday. We performed multivariable marginal Cox models to estimate the effect of off-hours (*versus* on-hours) on major catheter-related infections (MCRI) and catheter-related bloodstream infections (CRBSIs).

**Results:**

We included 7241 patients in 25 different ICUs, and 15,208 catheters, including 7226 and 7982 catheters inserted during off- and on-hours, respectively. Catheters inserted during off-hours were removed after 4 days (IQR 2, 9) in median, whereas catheters inserted during on-hours remained in place for 6 days (IQR 3,10; *p* < 0.01) in median. Femoral insertion was more frequent during off-hours. Among central venous catheters and after adjusting for well-known risk factors for intravascular catheter infection, we found a similar risk between off- and on-hours for MCRI (HR 0.91, 95% CI 0.61–1.37, *p* = 0.65) and CRBSI (HR 1.05, 95% CI 0.65–1.68, *p* = 0.85). Among central venous catheters with a dwell-time > 4 or > 6 days, we found a similar risk for MCRI and CRBSI between off- and on-hours. Similar results were observed for ACs.

**Conclusions:**

Off-hours did not increase the risk of intravascular catheter infections compared to on-hours. Off-hours insertion is not a sufficient reason for early catheter removal, even if femoral route has been selected.

**Supplementary Information:**

The online version contains supplementary material available at 10.1186/s13613-021-00940-3.

## Background

Intravascular catheters are instrumental in the care of intensive care unit (ICU) patients to allow safe intravenous administration of medications, enable the intravenous administration of fluid resuscitation and the monitoring of hemodynamic parameters. On one hand, the central venous catheter utilization rate is high with an average of 70.1 catheter-days per 100 patient days reported in European ICU [1]. On the other hand, ICU-bloodstream infections (BSIs) are reported as catheter-related in one-fourth to one-third of cases [2]. Intravascular catheter-related infections are associated with increased costs, morbidity and mortality [3, 4]. A large proportion of intravascular catheter infection is preventable [5].

Patients admitted to hospital during off-hours (i.e*.*, during the night or at weekend) may experience poorer quality of care and clinical outcomes due to the reduced human resources. For example, mortality was higher in acute myocardial infarct patients admitted during weekend daytime hours when compared with patients admitted during other times [6]. The degree to which intravascular catheter infections were associated with weekend or night-time (i.e*.*, off-hours) insertions reflects poorer quality of care remains an open issue. To our knowledge, no study has investigated the variations in clinical processes and risk of intravascular catheter infections between on- compared with off-hours insertions.

Our primary aim was to describe differences between patients and catheters inserted during on- *versus* off-hours. Our secondary aim was to investigate whether insertions during off-hours influenced the level of intravascular infectious risks.

## Material and methods

### Design

We performed a post hoc analysis using the databases from four large randomized-controlled trials (RCTs; i.e*.*, DRESSING1, DRESSING2, ELVIS and CLEAN). A prospective high-quality data collection was performed [7–10]. The similarities among all these RCTs concerning inclusion criteria and definitions allowed us to merge the four databases. The DRESSING1 investigated the effect of chlorhexidine gluconate (CHG) sponge-dressing for preventing intravascular catheter infections. The DRESSING2 study assessed the effect of CHG gel-dressing and highly adhesive dressing for preventing catheter-related infections and catheter colonization. The ELVIS study investigated the impact of intravascular catheter infection of preventive ethanol-based lock therapy in short-term dialysis catheters. The CLEAN study evaluated differences in infectious complications between skin antisepsis either with 2% alcoholic CHG and povidone iodine–alcohol [PVI]. CHG-dressings (i.e., sponge- or gel-dressing) and CHG-skin antisepsis decreased the risk of infection. However, ethanol-based lock did not reduce intravascular catheter infections. The study interventions were neither blinded to the ICU staff nor to the investigators; however, they were blinded to the microbiologists who processed the samples of skin, blood and catheter cultures and to the adjudication committee (see definitions). The current analysis complied with the STROBE guidelines for observational studies. The studies were approved by national ethic committees; further ethical consent was not required according to the French law for research.

### Patients

Patients older than 18 years were recruited from 2008 to 2014 in French ICU as soon as they required central venous catheters, a short-term dialysis catheter (DC) or peripheral arterial (AC) catheter insertion. The characteristics of included patients were similar across studies. Patients underwent follow-up until death or 48 h after ICU discharge.

### Catheters

This analysis evaluated data from patients with short-term central venous catheters, ACs and DCs included in the four RCTs. Catheters without exhaustive information on time of insertion were excluded. All catheters were managed in the same way. Investigators complied with French recommendations for catheter insertion and care, which are similar to CDC guidelines [11] (Additional file 1), which are described elsewhere [12]. Of note, prevention strategies did not substantially change since 2014 [13]. Importantly, randomization process was carried out immediately before catheter insertion. Information on insertion time was routinely collected in all RCTs. Time of catheter removal catheters was decided by the attending physician caring for each patient.

### Definitions

We used French definitions for intravascular catheter colonization and infections [14]. Catheter tip colonization was defined as a quantitative culture yielding ≥ 1000 cfu/mL [15]. A catheter-related bloodstream infection (CRBSI) was a combination of (1) one or more positive peripheral blood cultures sampled after at least 48 h of catheterization or maximal 48 h after catheter removal; (2) a blood culture differential time-to positivity of 2 h or more [16], or the isolation of the same phenotypic microorganism from the colonized catheter and (3) no apparent source of bloodstream infection (BSI) other than the catheter [7–10]. Catheter-related clinical sepsis without BSI was a combination of catheter colonization, body temperature, pus at the insertion site, or resolution of clinical sepsis after catheter removal, and the absence of any other infectious focus [17]. Major catheter-related infection (MCRI) was defined as either a CRBSI, or a catheter-related clinical sepsis without bloodstream infection. If a patient had a positive blood culture for coagulase-negative staphylococci (CoNS), two separate peripheral blood cultures had to grow the same microorganism that colonized the catheter tip. Alternatively, the same pulsotype from the strains recovered from the catheter tip and blood culture was required for a diagnosis of a CoNS-CRBSI. All suspected cases of catheter-related infections were reviewed by masked independent assessors based on detailed pre-established definitions [7–10].

As time of catheter insertion was available, we created a variable for night and weekend insertions (“off-hours” or “not regular business hours”). Of note, in France, off-hours started at 6 PM until 8:30 A.M. during the week. At weekend, we defined off-hours from 1 P.M. on Saturday to 8.30 A.M. on Monday. To ease the readability of manuscript, we simplified this variable in off- or on-hours.

During off-hours, in all ICUs, one senior physician (for a maximum of 20 ICU beds) and one resident are on duty. During on-hours, senior physician-to-patient ratio ranges from 1:3 to 1:6 and the resident-to-patient ratio ranges from 1:2 to 1:5.

Skin colonization at insertion site colonization at the time of catheter removal was evaluated in three studies using semi-quantitative insertion-site cultures: the insertion site was sampled immediately before catheter removal [7, 8, 10]. As previously analyzed [12, 18] and because the size of the insertion site cultured was different across studies, we created a semi-quantitative variable with sterile, low-grade colonization, and high-grade colonization according to the median of quantitative cultures obtained in each study.

### Statistical analysis

Characteristics of patients and catheters were described as median (interquartile range, IQR) or count (percent) for quantitative and qualitative variables, respectively. For group comparison, we used Chi-square or Fisher and Wilcoxon tests as appropriate.

We performed marginal Cox models for clustered data, in order to take into account a possible clustering effect of multiple catheters per patient and stratifying by center. Data were censored at 28 days after catheter insertion. Hazard ratio (HR) for MCRI, CRBSI or catheter tip colonization was evaluated by multivariate analysis. The variable off-hours (*versus* on-hours) was forced in the multivariate models and other relevant well-known intravascular catheter infection risk factors were used as adjustment variables. The following adjustment variables were selected: gender, SAPS II, insertion site, experience of the operator, skin antisepsis (alcoholic chlorhexidine gluconate [CHG] *versus* povidone iodine), CHG-impregnated dressings (either sponge- or gel-dressings *versus* non-impregnated dressings), time from ICU admission to catheter insertion, mechanical ventilation and vasopressor at insertion. The effect of “off-hours” on MCRI, CRBSI and catheter tip colonization was estimated. A hazard ratio (HR) > 1 indicated an increased risk for off-hours compared to on-hours. This marginal Cox model we used considers the intra-cluster dependency (i.e*.*, more than one catheter per patient), using robust sandwich covariance estimates (PROC PHREG of SAS) [19]. The proportionality of hazard for off-hours was tested using Martingale residuals. Analyses were separated for central venous catheters and ACs. We pooled both central venous catheters and DCs in the same variable (i.e*.*, CVC). We performed several additional analyses: (1) we analyzed the risk of intravascular catheter infections between off- and on-hours for catheters inserted more than 4 days or 6 days; (2) we assessed whether femoral insertions during off-hours were associated with increased risk in non-subclavian catheters; (3) we performed a sensitivity analysis excluding the first inserted intravascular central venous catheter or excluding patients admitted for planned surgery using MCRI as an outcome. Tests were two-tailed, with *p* < 0.05 being considered significant. All analyses were performed using SAS (version 9.4). Further details on randomization groups or missing data were available in the Additional file 1.

## Results

### Patients and catheters

**Fig. 1:**
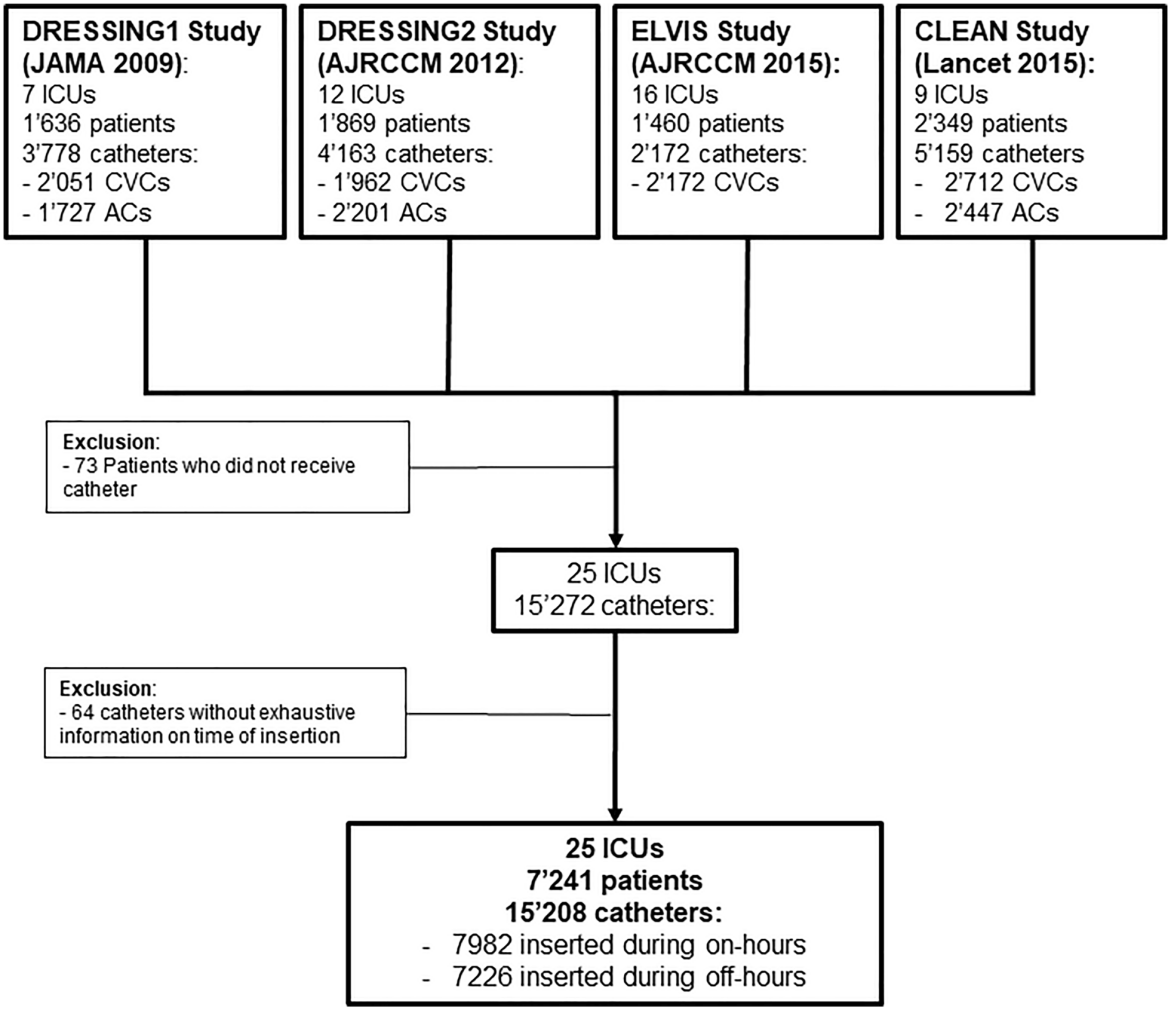
Flowchart. ICU: intensive care unit. CVC: central venous catheters and short-term dialysis catheters. AC: arterial catheter

Between 2009 and 2014, we included 7241 patients and 15,208 catheters from 25 different ICUs (Fig. 1). We monitored 6338 ACs, 6142 central venous catheters and 2,28 DCs.

Catheters inserted during off- and on-hours were 7226 and 7982, respectively. Characteristics of patients and catheters are illustrated in Tables 1 and 2. Overall, patients with catheter insertions during on-hours were similar to patients with off-hours insertions. Catheters inserted in solid organ transplant patients were more frequently inserted during on-hours (4.5% *versus* off-hours 3.3%, *p* = 0.02). ICU mortality was similar between both groups.

**Table 1:** Patients characteristics (*n* = 5548)

		On-hours	Off-hours	*p*-value*
Sex	Female	996 (35.4)	1,007 (36.8)	0.27
Age, median [IQR]		64 [53; 74]	65 [53; 75]	0.06
Without comorbidity		1,602 (56.9)	1,586 (58)	0.43
Chronic renal failure		143 (5.1)	129 (4.7)	0.53
Chronic heart failure		208 (7.4)	199 (7.3)	0.87
Diabetes		225 (8)	224 (8.2)	0.79
Chronic respiratory failure		153 (5.4)	140 (5.1)	0.59
HIV		65 (2.3)	57 (2.1)	0.57
Solid organ transplant		127 (4.5)	91 (3.3)	0.02
Other immunosuppression		176 (6.3)	158 (5.8)	0.45
Hematological malignancy		151 (5.4)	146 (5.3)	0.96
Reason for ICU admission	Septic shock	590 (21)	619 (22.6)	< 0.01
	Planned surgery	126 (4.5)	60 (2.2)	
	Trauma	148 (5.3)	142 (5.2)	
	Abdominal MOF	96 (3.4)	77 (2.8)	
	Cardiac shock	208 (7.4)	266 (9.7)	
	Hemorrhagic shock	125 (4.4)	125 (4.6)	
	Shock (other)	73 (2.6)	83 (3)	
	Respiratory failure	666 (23.7)	540 (19.7)	
	COPD exacerbation	65 (2.3)	50 (1.8)	
	Renal failure	191 (6.8)	253 (9.3)	
	Coma	266 (9.5)	280 (10.2)	
	Continuous surveillance	259 (9.2)	240 (8.8)	
Mechanical ventilation in the first 24 h		2037 (72.4)	1948 (71.2)	0.33
SAPS II, median [IQR]		54 [39; 69]	54 [41; 71]	0.07
SOFA score, median [IQR]		10 [7; 14]	10 [7; 14]	0.53
ICU mortality		909 (32.3)	905 (33.1)	0.54
ICU length of stay, median [IQR]		9 [5; 18]	7 [4; 13]	< 0.01

1,693 patients had both insertion during the night and day and were excluded from this analysis. Data were expressed in n (percentage) or median [interquartile range]. * Without adjustment

**Table 2:** Catheters characteristics (*n* = 15,208)

Catheters (*n* = 15,208)		On-hours	Off-hours	*p*-value**
Catheter-days, median [IQR]		6 [3; 10]	4 [2; 9]	< 0.01
First catheter		3494 (43.8)	3661 (50.7)	< 0.01
Experience level of the operator	< 50 procedures	5184 (64.9)	4101 (56.8)	< 0.01
Insertion site for AC	Femoral	1076 (33.4)	1176 (37.7)	< 0.01
	Radial	2144 (66.6)	1942 (62.3)	
Insertion site for central venous catheter	Jugular	1084 (33.6)	875 (30)	< 0.01
	Subclavian	1264 (39.1)	951 (32.6)	
	Femoral	881 (27.3)	1087 (37.3)	
Insertion site DC	Jugular	581 (37.9)	327 (27.4)	< 0.01
	Subclavian	24 (1.6)	11 (0.9)	
	Femoral	928 (60.5)	857 (71.7)	
Catheter type for central venous catheter	CVC	3229 (67.8)	2913 (70.9)	< 0.01
	DC	1533 (32.2)	1195 (29.1)	
Ultrasound guidance*		857 (17.9)	695 (16.1)	0.02
CHG-impregnated dressings		2055 (25.7)	1991 (27.6)	0.01
Skin antisepsis with CHG		3640 (45.6)	3263 (45.2)	0.58
Mechanical ventilation at insertion		6108 (76.5)	5399 (74.7)	< 0.01
Vasopressor at insertion		4138 (51.8)	3958 (54.8)	< 0.01
Antibiotics at insertion		5171 (64.8)	4396 (60.8)	< 0.01
Reason for removal	Death	1730 (21.7)	1649 (22.8)	0.09
	No longer needed	2371 (29.7)	2277 (31.5)	0.02
	Suspicion of infection	1133 (14.2)	881 (12.2)	< 0.01
	ICU discharge	1728 (21.6)	1647 (22.8)	0.09
	Bleeding	18 (0.2)	8 (0.1)	0.09
	Wrenched	111 (1.4)	103 (1.4)	0.86
	Dysfunction***	711 (11.7)	584 (10.9)	0.19
MCRI		88 (1.1)	67 (0.9)	0.28
CRBSI		60 (0.8)	48 (0.7)	0.52
Catheter tip colonization		653 (8.2)	540 (7.5)	0.10

^*^Missing information in 6107 catheters (i.e*.*, DRESSING1 and ELVIS studies). Data were expressed in n (percentage) or median [interquartile range]. ** Without adjustment. *** This information was available only for the DRESSING2, CLEAN and ELVIS study but not for the DRESSING1 study (total missing data n = 3778)

Catheters inserted during off-hours were removed after 4 days (IQR 2, 9) in median, whereas catheters inserted during on-hours remained in place for 6 days (IQR 3,10; *p* < 0.01) in median. Dwell-time among first inserted catheters were again shorter in the off-hours group (4 days, IQR 2, 8) compared to the on-hours group (5 days [IQR 2, 9], *p* < 0.01). Junior operators (i.e*.*, < 50 procedures) inserted intravascular catheters more frequently during on-hours (64.9%) compared to off-hours (56.8%, *p* < 0.01). The choice of insertion site changed between on- and off-hours, with femoral insertions being more frequent during off-hours for AC, central venous catheter and DC. Interestingly, no major percentage (less than 2.2%) differences in reasons for catheter removal were observed between the two groups. Suspicion of infection were more frequently observed in catheters inserted during on-hours (14.2% *versus* off-hours 12.2%, *p* < 0.01). We observed 88 (1.1% or 1.50 per 1000 catheter-days), 60 (0.8% or 1.02 per 1000 catheter-days) and 653 (8.2% or 11 per 1000 catheter-days) MCRI, CRBSI and catheter tip colonization in the on-hours group, respectively. We observed 67 (0.9% or 1.47 per 1000 catheter-days), 48 (0.7% or 1.02 per 1000 catheter-days) and 540 (7.5% or 12 per 1000 catheter-days) MCRI, CRBSI and catheter tip colonization during off-hours, respectively. No differences in MCRI, CRBSI and catheter tip colonization prevalence were observed between the two groups.

### Infectious risk for off-hours in CVCs

**Fig. 2:**
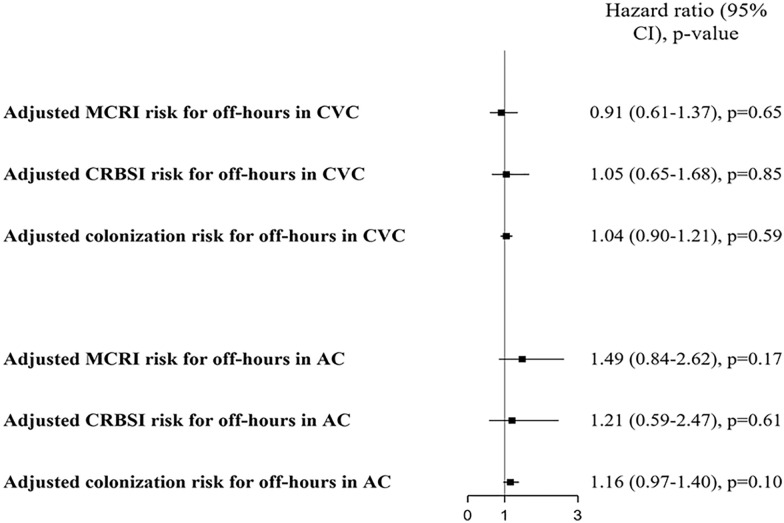
Adjusted MCRI, CRBSI and colonization hazard risk for off-hours in CVCs and ACs. CI: confidence interval. MCRI: major catheter-related bloodstream infection. CRBSI: catheter-related bloodstream infection. CVC: central venous catheter. AC: arterial catheter. A hazard ratio (HR) > 1 indicated an increased risk for off-hours compared to on-hours. Adjustment variables were the following: gender, SAPS II, insertion site, experience of the operator, skin antisepsis, CHG-impregnated dressings, time from ICU admission to catheter insertion, mechanical ventilation and vasopressor at insertion

Among CVCs and after adjustment for well-known risk factors for intravascular catheter infection, we found a similar risk between off- and on-hours for MCRI (HR 0.91, 95% CI 0.61–1.37, *p* = 0.65), CRBSI (HR 1.05, 95% CI 0.65–1.68, *p* = 0.85) and catheter tip colonization (HR 1.04, 95% CI 0.90–1.21, *p* = 0.59, Fig. 2). Among CVCs with a dwell-time > 4 days, we found a similar risk between off- and on-hours for MCRI (HR 0.82, 95% CI 0.52–1.2, *p* = 0.39), CRBSI (HR 0.92, 95% CI 0.53–1.59, *p* = 0.76) and catheter tip colonization (HR 0.97, 95% CI 0.81–1.16, *p* = 0.74). Among CVCs with a dwell-time > 6 days, we found a similar risk between off- and on-hours for MCRI (HR 0.77, 95% CI 0.47–1.27, *p* = 0.31), CRBSI (HR 0.76, 95% CI 0.42–1.39, *p* = 0.38) and catheter tip colonization (HR 0.97, 95% CI 0.79–1.20, *p* = 0.79). Among non-subclavian CVCs inserted during off-time, the femoral site was not associated with an increased risk of MCRI (HR 0.97, 95% CI 0.48–1.96, *p* = 0.94) and CRBSI (HR 0.92, 95% CI 0.43–1.96, *p* = 0.83). However, the risk of catheter tip colonization for femoral insertions was increased compared to jugular insertions (HR 1.57, 95% CI 1.20–2.07, *p* = 0.0012) during off-hours.

Sensitivities analyses excluding the first inserted CVC or excluding patients admitted for planned surgery using MCRI as an outcome are illustrated in the Additional file 1 and showed similar results.

### Infectious risk for off-hours in ACs

After adjustment for well-known risk factors for intravascular catheter infection, the level of risk was similar between off- and on-hours for MCRI (HR 1.49, 95% CI 0.84–2.62, *p* = 0.17), CRBSI (HR 1.21, 95% CI 0.59–2.47, *p* = 0.61) and catheter tip colonization (HR 1.16, 95% CI 0.97–1.40, *p* = 0.10, Fig. 2). Among ACs with a dwell-time > 4 days, the level of risk was also similar between off- and on-hours for MCRI (HR 1.50, 95% CI 0.79–2.84, *p* = 0.21), CRBSI (HR 1.49, 95% CI 0.69–3.23, *p* = 0.31) and catheter tip colonization (HR 1.10, 95% CI 0.89–1.36, *p* = 0.39). Among ACs with a dwell-time > 6 days, the level of risk was also similar (data not shown). Among ACs inserted during off-time, the femoral site was not associated with an increased risk of MCRI (HR 0.85, 95% CI 0.31–2.33, *p* = 0.75) and CRBSI (HR 0.61, 95% CI 0.17–2.15, *p* = 0.44). Catheter tip colonization risk was increased for femoral insertions compared to radial insertions (HR 1.74, 95% CI 1.31–2.31, *p* = 0.0001) during off-hours.

### Skin colonization at catheter removal between on- and off-hours

Skin colonization at insertion site colonization at the time of catheter removal (variable available for 9478 catheters) was more frequently colonized in the on-hours group compared to the off-hours (*p* < 0.01, Table 3). Considering only catheters with ≤ 4 days of maintenance (*n* = 4129), no significant difference was observed between on- and off-hours groups (*p* = 0.11).

**Table 3:** Skin colonization at catheter removal

	On-hours	Off-hours	*p*-value
Skin colonization at removal (*n* = 9478)			
High-grade colonization	1520 (31.2)	1328 (28.9)	< 0.01
Low-grade colonization	1429 (29.3)	1302 (28.3)	
Sterile	1929 (39.5)	1970 (42.8)	
Skin colonization at removal, ≤ 4 catheter-days (*n* = 4129)			
High-grade colonization	441 (23.2)	475 (21.3)	0.11
Low-grade colonization	570 (30)	642 (28.8)	
Sterile	887 (46.7)	1114 (49.9)	
Skin colonization at removal, > 4 catheter-days (*n* = 5349)			
High-grade colonization	1079 (36.2)	853 (36)	0.62
Low-grade colonization	859 (28.8)	660 (27.9)	
Sterile	1042 (35)	856 (36.1)	

## Discussion

Using high-quality data from four RCTs, this post hoc analysis showed that intravascular catheters inserted during off-hours were removed earlier on, and were more frequently inserted in femoral sites compared to catheters inserted during on-hours. Off-hours insertions did not increase the risk of intravascular catheter infections and the femoral site did not substantially increase the infectious risk during off-hours. Although several outcomes (i.e*.*, mortality, surgical site infections) according to the time of admission or intervention were assessed [20–23], to our knowledge, this is the first study that addressed this topic for intravascular catheter infections.

Catheters removal was performed *earlier* if inserted during off-hours. Reasons for removal between on- and off-hours showed similar percentages. We found a decreased dwell-time for off-hours catheters after exclusion of the first insertions or patients admitted for planned surgery. Interestingly, this finding was not related to a more frequent suspicion of infection at the time of catheter removal. We therefore suppose that catheters inserted during off-hours were more frequently removed for clinician’s *fear* of possible contamination. However, we cannot exclude a longer administration of catecholamine for catheters inserted during on-hours. Interestingly, skin at exit site at removal in catheters inserted during off-hours was less colonized compared to catheters inserted during on-hours, thus possibly reflecting a premature removal for off-hours-inserted catheters. Similar results were observed for skin colonization at insertion site in catheters with short duration of catheter maintenance (i.e*.*, < 4 days), probably reflecting a similar extraluminal contaminations between both groups. Importantly, comparing catheters with long duration (i.e*.*, > 4 or 6 days) we did not detect any differences in infectious risk between on- and off-hours. In light of these considerations, we believe that catheters inserted during off-hours should be managed in a similar way as catheters inserted during on-hours and should probably not be removed early.

Intravascular catheters were more frequently inserted in the femoral vein or artery during off-hours. Interestingly, after excluding subclavian catheters (i.e*.*, insertion site with well-established reduced infectious risk), neither arterial nor venous femoral site increased the intravascular catheter risk of infection (MCRI and CRBSI) during off-hours. However, the catheter tip colonization risk increased in femoral inserted catheters. This result should be interpreted with caution. We described that catheter tip colonization showed poor agreement with intravascular catheter infections (i.e*.*, CRBSI) and, probably, catheter tip colonization reflected an unsuitable surrogate marker for intravascular catheter infections [24]. In light of these results, we discourage routine replacement of intravascular catheters inserted in the femoral site during off-hours.

Our analysis has several limitations. First, we performed an observational study of prospectively collected RCT data, patients could not be randomized according to our interest variable and unmeasured factors may persist, thus causing residual confounding. However, this high-quality database allowed us to adjust for several confounders. Second, infection prevention and control (IPC) measures in patients included in RCT were probably better implemented than IPC measures under real-life conditions. It is therefore conceivable that results may differ when considering patients not included in RCTs. Third, the original RCTs were designed to investigate the impact of certain infection prevention measures, and interactions may have occurred among the study groups or centers. However, our statistical analyses considered these potential drawbacks and our models were stratified by centers. Fourth, our interest variable (on- *versus* off-hours) was based on French policies, which may limit the generalizability of our results to other countries. Fifth, we could analyze at catheter level the impact of staffing (e.g*.*, resident or advanced practice providers alone without senior physicians) on intravascular catheter infections. On the other hand, intravascular catheters were inserted by more experienced operators during off-hours, thus mitigating the impact of off-hours on the main results. However, our models considered the experience of the operator as adjustment factor.

## Conclusions

In ICUs where catheter-infection prevention measures are fully implemented, insertion during off-hours was not associated with an increased risk of infections compared to catheters inserted during on-hours. Off-hours insertion is not a sufficient reason for early catheter removal, even if femoral route has been selected.

## Supplementary Information

**Additional file 1.** Practices and intravascular catheter infection during on- and off-hours in critically ill patients.

## Data Availability

The datasets used and/or analyzed during the current study are available from the corresponding author on reasonable request.

## Abbreviations

AC: Peripheral arterial catheter.
CoNS: Coagulase-negative staphylococci.
CRBSI: Catheter-related bloodstream infections.
CVC: Central venous catheter and short-term dialysis catheter.
DC: Short-term dialysis catheter
HR: Hazard ratio.
ICU: Intensive care unit.
IPC: Infection prevention and control.
IQR: Interquartile range.
MCRI: Major catheter-related infection.
RCT: Randomized controlled-trial.
SOFA: Sepsis-related Organ Failure Assessment
