# Supplementary material for: Practices and intravascular catheter infection during on- and off-hours in critically ill patients
Source: Ann Intensive Care. 2021 Oct 29;11:153. doi: 10.1186/s13613-021-00940-3 (PMC8556470; doi:10.1186/s13613-021-00940-3)
Supplement: Supplementary file 1 — Additional file 1. Practices and intravascular catheter infection during on- and off-hours in critically ill patients. [file 13613_2021_940_MOESM1_ESM.docx]

**Additional file 1**

**Supplementary methods**

Merging process and differences among the studies. The variables’ and outcomes’ definitions in the four studies were the same. For the DRESSING1 and ELVIS studies, no information on ultrasound guidance was available. Skin culture results at removal were not available in the ELVIS study. The variable skin antisepsis was simplified in CHG (without considering the percentage of CHG or alcohol) *versus* non-CHG.

French recommendations for catheter insertion and care. 1) Maximal sterile barrier precautions were used (large sterile drape; surgical hand antisepsis; and mask, cap, sterile gloves, and gown). 2) The site of insertion was selected according to the discretion of the physician caring for the patient. 3) PVI or CHG was used for skin antisepsis at catheter insertion and during dressing changes according to discretion of the physician or to randomisation scheme. 4) Semipermeable or CHG-impregnated dressings were used at all insertion sites and were changed 24 hours after catheter insertion and then every 3 or 7 days according to standard practice in each ICU or to randomisation scheme. Leaking, soiled, or wet dressings were changed immediately. None of the study catheters was antibiotic-impregnated or antiseptic-impregnated.

Randomization groups and statistical analysis. CHG dressings (interventions in DRESSING1 and DRESSING2 studies) were used as adjustment covariates. The covariate “CHG skin disinfection” (i.e., intervention in the CLEAN study) was used as adjustment covariate in the multivariate Cox models. Since ethanol-based lock did not influence intravascular catheter infections, we did not include this variable in our models.

Missing data. For all RCTs, in patients who needed to keep the CVC beyond ICU discharge, paired blood samples were drawn simultaneously from the catheter hub and a peripheral vein before ICU discharge for determination of the differential time-to-positivity.

**Supplementary results**

Sensitivity analysis excluding the first inserted central venous catheter. The analysis of only subsequent catheterizations showed that the median dwell-time was decreased for off-hours (5 days [3 ; 10] *versus* 7 days [3 ; 11] for on-hours, p<0.01). After adjusting for well-known risk factors for intravascular catheter infection, the level of risk was similar between off- and on-hours for MCRI (HR 0.96, 95% CI 0.55-1.70, p=0.90).

Sensitivity analysis excluding patients admitted for planned surgery. The median dwell-time was decreased for off-hours (5 days [2 ; 9] versus 6 days [3 ; 10] for on-hours p<0.01). After adjustment for well-known risk factors for intravascular catheter infection, the level of risk was similar between off- and on-hours for MCRI (HR 0.88, 95% CI 0.58-1.31, p=0.52).
